# Impact of a Mobile App on Paramedics’ Perceived and Physiologic Stress Response During Simulated Prehospital Pediatric Cardiopulmonary Resuscitation: Study Nested Within a Multicenter Randomized Controlled Trial

**DOI:** 10.2196/31748

**Published:** 2021-10-07

**Authors:** Matthieu Lacour, Laurie Bloudeau, Christophe Combescure, Kevin Haddad, Florence Hugon, Laurent Suppan, Frédérique Rodieux, Christian Lovis, Alain Gervaix, Frédéric Ehrler, Sergio Manzano, Johan N Siebert, PedAMINES Prehospital Group

**Affiliations:** 1 University of Geneva Faculty of Medicine Geneva Switzerland; 2 A.C.E. Geneva Ambulances SA Geneva Switzerland; 3 Division of Clinical Epidemiology, Department of Health and Community Medicine Geneva University Hospitals Geneva Switzerland; 4 Department of Pediatric Emergency Medicine, Geneva Children’s Hospital Geneva University Hospitals Geneva Switzerland; 5 Department of Emergency Medicine Geneva University Hospitals Geneva Switzerland; 6 Division of Clinical Pharmacology and Toxicology, Department of Anesthesiology, Clinical Pharmacology, Intensive Care and Emergency Medicine Geneva University Hospitals Geneva Switzerland; 7 Division of Medical Information Sciences, Department of Radiology and Medical Informatics Geneva University Hospitals Geneva Switzerland; 8 See Authors’ Contributions

**Keywords:** cardiopulmonary resuscitation, drugs, emergency medical services, medication errors, mobile health, mobile apps, out-of-hospital cardiac arrest, paramedics, pediatrics, State-Trait Anxiety Inventory, stress

## Abstract

**Background:**

Out-of-hospital cardiac arrests (OHCAs) are stressful, high-stake events that are associated with low survival rates. Acute stress experienced in this situation is associated with lower cardiopulmonary resuscitation performance in calculating drug dosages by emergency medical services. Children are particularly vulnerable to such errors. To date, no app has been validated to specifically support emergency drug preparation by paramedics through reducing the stress level of this procedure and medication errors.

**Objective:**

This study aims to determine the effectiveness of an evidence-based mobile app compared with that of the conventional preparation methods in reducing acute stress in paramedics at the psychological and physiological levels while safely preparing emergency drugs during simulated pediatric OHCA scenarios.

**Methods:**

In a parent, multicenter, randomized controlled trial of 14 emergency medical services, perceived and physiologic stress of advanced paramedics with drug preparation autonomy was assessed during a 20-minute, standardized, fully video-recorded, and highly realistic pediatric OHCA scenario in an 18-month-old child. The primary outcome was participants’ self-reported psychological stress perceived during sequential preparations of 4 intravenous emergency drugs (epinephrine, midazolam, 10% dextrose, and sodium bicarbonate) with the support of the PedAMINES (Pediatric Accurate Medication in Emergency Situations) app designed to help pediatric drug preparation (intervention) or conventional methods (control). The State-Trait Anxiety Inventory and Visual Analog Scale questionnaires were used to measure perceived stress. The secondary outcome was physiologic stress, measured by a single continuous measurement of the participants’ heart rate with optical photoplethysmography.

**Results:**

From September 3, 2019, to January 21, 2020, 150 advanced paramedics underwent randomization. A total of 74 participants were assigned to the mobile app (intervention group), and 76 did not use the app (control group). A total of 600 drug doses were prepared. Higher State-Trait Anxiety Inventory–perceived stress increase from baseline was observed during the scenario using the conventional methods (mean 35.4, SD 8.2 to mean 49.8, SD 13.2; a 41.3%, 35.0 increase) than when using the app (mean 36.1, SD 8.1 to mean 39.0, SD 8.4; a 12.3%, 29.0 increase). This revealed a 30.1% (95% CI 20.5%-39.8%; *P*<.001) lower relative change in stress response in participants who used the app. On the Visual Analog Scale questionnaire, participants in the control group reported a higher increase in stress at the peak of the scenario (mean 7.1, SD 1.8 vs mean 6.4, SD 1.9; difference: −0.8, 95% CI −1.3 to −0.2; *P*=.005). Increase in heart rate during the scenario and over the 4 drugs was not different between the 2 groups.

**Conclusions:**

Compared with the conventional method, dedicated mobile apps can reduce acute perceived stress during the preparation of emergency drugs in the prehospital setting during critical situations. These findings can help advance the development and evaluation of mobile apps for OHCA management and should be encouraged.

**Trial Registration:**

ClinicalTrials.gov NCT03921346; https://clinicaltrials.gov/ct2/show/NCT03921346

**International Registered Report Identifier (IRRID):**

RR2-10.1186/s13063-019-3726-4

## Introduction

### Background

Out-of-hospital cardiac arrest (OHCA) is a major concern for health care systems worldwide, affecting millions of people each year [1]. Despite advances in resuscitation science and improvement of cardiac arrest survival over the past decades, the survival rates following adult and pediatric OHCA are reportedly low, evaluated at 10.4% and 11.4%, respectively [1]. High-quality cardiopulmonary resuscitation (CPR) for OHCA patients is the primary determinant of survival and favorable neurological outcome [2,3]. Evaluations and decisions must be made quickly and accurately. However, acute mental stress experienced by rescuers during CPR may impair decision making and optimal performance [4-12], independent of professional experience [4]. This can, in turn, adversely affect patient safety [5]. OHCA-induced acute stress response relies mainly on an interplay between the individual's cognitive perception and appraisal made about the perceived demand and ability to compensate through both internal and environmental resources [6,13]. The degree to which this compensation occurs determines the nature and magnitude of one's stress response [12]. Some individuals show stress responses with associated active coping. Others perceive stress as excessive and outweighing their coping abilities, thus hindering their ability to adapt quickly and perform under pressure.

The OHCA setting is a stressful and high-stakes environment where safeguards and resources, both human and material, are limited [6]. In many countries, paramedics have the autonomy to prepare and administer emergency drugs. However, the impact of acute stress experienced by paramedics during OHCA on emergency drug preparation has rarely been studied. LeBlanc et al [14] observed that paramedics under simulated high-stress conditions performed worse on drug dosage calculations than those under calm, relaxed conditions. These findings are particularly concerning in pediatric CPR, where the accurate and safe preparation and administration of intravenous drugs is mandatory [15-19]. Most drugs administered intravenously to children are provided in vials that were originally prepared for the adult population. This leads to the need for an initial onsite complicated, individual, weight-based dose calculation, and drug preparation for each child, which varies widely across age groups [20]. Combined with other risk factors such as excessive extraneous cognitive load due to onsite emotional stress and time pressure [14,21-23], and pediatric-specific, age-related variations in pharmacokinetics, onsite administration of emergency drugs by paramedics is particularly challenging. Furthermore, pediatric situations only account for approximately 7% of emergency medical services (EMS) calls, and paramedics have little exposure to critically ill children and occasions to prepare emergency drugs at pediatric dosages [24-26]. Relying solely on their expertise and knowledge to take decisions during care provision, a single paramedic is often in charge of determining the child’s weight, choosing the most suitable drug, calculating the drug dose and appropriate volume to inject, and administering it without delay. For this purpose, paramedics are still dependent on conventional paper-based support, empirical calculators, or spreadsheets to ensure correct drug delivery. This places children at higher risk for life-threatening prehospital medication errors than adults [17,20,27-30], with a reported error rate of more than 60% [31,32].

Some authors have advocated replacing tasks inducing stress and cognitive load during resuscitation as much as possible by automated actions to optimize patient care and diminish medication errors [22,33]. The US National Highway Traffic Safety Administration advocated, in its recent vision for the future of pediatric prehospital care to be achieved by 2050, to develop processes that do not require providers to calculate dosing of medications [34]. Supported by the rapid spread of mobile devices and their innovative features (eg, connectivity, embedded computing capabilities, small size, and versatility), mobile health (mHealth) apps have great potential as tools to support out-of-hospital emergency drug preparation at the point of care. However, a recent systematic review showed that few mHealth apps are available for prehospital settings [35]. Among these mHealth apps, none has been validated to specifically support emergency drug preparation by EMS personnel with the aim of reducing medication dosing errors and the stress hassle of this procedure.

### Previous Work

In previous randomized trials, we reported fewer medication errors and shorter times to drug preparation and delivery during in-hospital pediatric CPR when using a mobile app—the PedAMINES (Pediatric Accurate Medication in Emergency Situations) app compared with conventional preparation methods [36,37]. Although similarities exist, the prehospital environment is distinctly different in many regards. Recent findings of a multicenter randomized trial showed that this app was also able to reduce medication error rates during pediatrics OHCA in a simulated model [38]. However, its impact on situational stress experienced by paramedics during CPR remains to be determined.

### Aim

This study aims to determine the effectiveness of the PedAMINES app in reducing acute stress while safely preparing emergency drugs during CPR for pediatric OHCA patients compared with the conventional preparation methods.

## Methods

### Study Design

This study was registered at ClinicalTrials.gov (NCT03921346) as a nested study within the context of an open-label, multicenter, randomized controlled trial. The parent trial had the broader and primary aim of assessing rates of medication dosing errors during simulation-based pediatric OHCA scenarios using a high-fidelity manikin [38]. The trial protocol, containing details about the scenario, has been previously published [39]. No changes were made to the app or intervention during the study.

### Trial Participants

The trial was conducted at 14 EMS covering a population of more than 2.3 million people in Switzerland. Eligible participants were registered paramedics working in these EMS. They had undergone a 3-year education program and were trained in advanced life support procedures, including defibrillation, airway management, peripheral intravenous line cannulation, and the administration of medications to ensure advanced independent emergency prehospital care. Similar to the Anglo-American model [40,41], paramedics in Switzerland constitute the initial response team and are qualified to independently administer a range of medications. To achieve adequate participant enrolment to reach the target sample size (ie, 120 paramedics [39]), shift-working paramedics were randomly recruited weeks before the start of the study by a blinded noninvestigator in each EMS center. In several EMS centers, additional paramedics were recruited to ensure that the target sample size would ultimately be achieved. At the end of the trial, an additional number of paramedics was included in the study and the analysis was refined. To prevent preparation bias, all paramedics were informed of the upcoming simulation study, but not of its purpose and outcomes. The study excluded emergency medical technicians because they had no drug preparation autonomy. In our study, resuscitation was led by a physician (JNS) to standardize the choice of drugs prescribed across the EMS and to avoid any deviation from the study protocol. Written informed consent was obtained from all the participants before their voluntary involvement. Blinding to the purpose of the trial during recruitment was maintained to minimize preparation bias. Participants were unblinded during the intervention when they were asked to prepare the drugs either with the support of the app or conventional methods. Study participants were neither involved in the design of the app, study design, choice of outcome measures, nor study conduct. No participant was asked to advice on the interpretation or write the results.

### Setting

The trial took place in a simulated, regular child’s bedroom environment at each EMS center. This was intended to mimic as closely as possible a stressful prehospital environment where paramedics could actually intervene to increase realism. The intervention was standardized across all sites to follow the same chronological progression and range of difficulty in order to ensure that each participant was exposed to exactly the same situation, with similar challenges in decision making and treatment preparation provided on the same manikin (Laerdal New SimBaby, Tetherless, Laerdal Medical, Stavanger, Norway). The study team members maintained a stressful resuscitation atmosphere. Importantly, we did not organize pretests to minimize priming and preparation biases to avoid influencing the perceived stress during the scenario.

### Intervention

On the day of the participation after random allocation, each participating paramedic had to complete 2 self-administered questionnaires to measure their perceived stress at baseline (see below), and a heart rate (HR) monitor was placed on their wrist. They also had to complete a survey collecting data regarding their demographics, attend a standardized 5-minute training session on how to use the mobile app, and follow a presentation detailing the features of the simulation manikin. No conventional drug preparation training was provided in either group, as this was part of the paramedics’ daily practice. Each participant was then exposed to a 20-minute, standardized, fully video-recorded, highly realistic pediatric OHCA CPR scenario of an 18-month-old child. The participants were asked to sequentially prepare and inject 4 different direct intravenous drugs of various degrees of preparation difficulties (epinephrine, midazolam, dextrose 10%, and sodium bicarbonate), either with the support of the app or following conventional methods (ie, without app support). Full details of the intervention and scenario have been previously published [38]. During the timed scenario, the resuscitation team maintained a stressful and realistic resuscitation atmosphere by frequently reporting vital signs aloud and asking the participant to promptly provide the drugs. The portable defibrillator, displaying real time vital signs, was placed in close proximity to the paramedics. The monitoring alarms were turned on, and the investigator who played the father’s role repeatedly verbalized his dismay.

### The PedAMINES Mobile App

**Figure 1:**
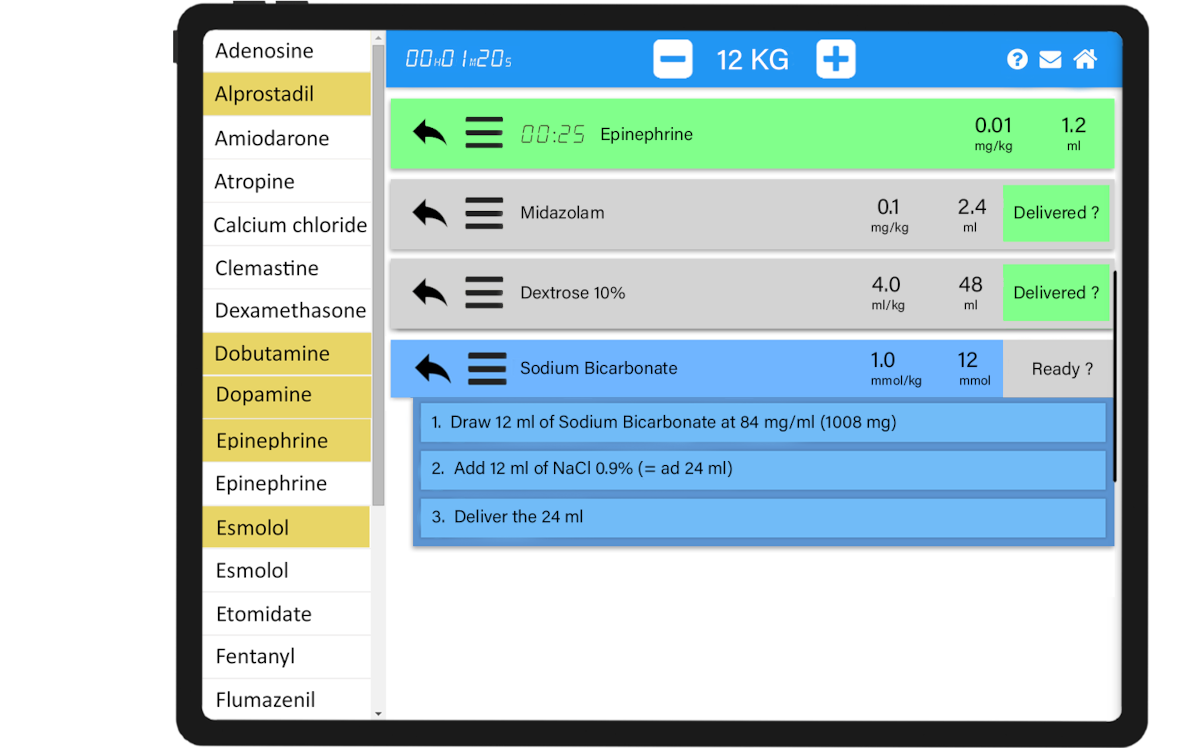
PedAMINES (Pediatric Accurate Medication in Emergency Situations) app screenshot.

This app provided an exhaustive and editable list of intravenous drugs, either for direct injections or continuous infusions [37,38,42]. The drugs were displayed in alphabetical order with doses automatically adapted to the weight or age of the patient. When selected, a detailed drug preparation procedure based on a standardized and simplified pathway was provided to the user. The app was developed using a user-centered, evidence-based approach. Emergency department caregivers, ergonomists, psychologists, and computer scientists from research and development services conceived the app. Its interface was designed considering design principles aimed at minimizing cognitive load [43-45]. By always displaying the most important information in a larger font and sorting the most recent information at the top of the screen, hierarchization of information was taken into consideration. Interaction choices were limited to their strictest utility through the interface to facilitate decision making. Only a predefined sequence of actions was proposed; thus, users did not need to make complicated choices. Display conventions were defined to display drug dosages with conventional and consistent units to avoid confusion. Fits laws were complied with by placing interaction buttons at the edge of the screen [46]. This minimized the distance to reach them and focused on the interaction zone in a limited area. The app also complied with the progressive disclosure principle [47], where complex information is sequenced across several smaller chunks to reduce the feeling of being overwhelmed by the user. Therefore, complex instructions such as weight-based drug dose preparation was sequenced and ordered in several lines to streamline information and facilitate its comprehension (Figure 1). Feedback mechanisms have also been integrated for specific actions, such as canceling or modifying the patient’s weight. In both cases, the system provides feedback to ensure that users are aware of their actions. Acceptance and use of the app were assessed through self-administrated electronic surveys using a modified version of the Unified Theory of Acceptance and Use of Technology (UTAUT) model [48] and the System Usability Scale [49,50]. They will be the subject of detailed analysis in another study nested within the parent trial.

### Outcomes and Measures

#### Primary Outcome: Perceived Stress

**Figure 2:**
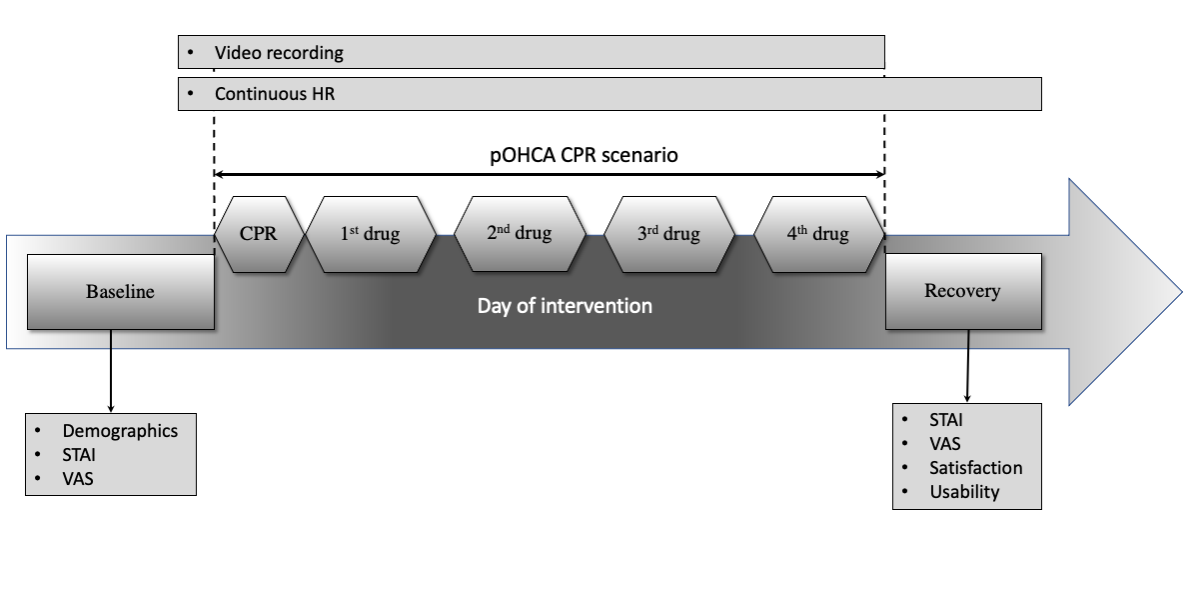
Course of the intervention. CPR: cardiopulmonary resuscitation; HR: heart rate; pOHCA: pediatric out-of-hospital cardiac arrest; STAI: State-Trait Anxiety Inventory; VAS: visual analogue scale.

Participants’ self-assessed psychological stress was measured using the Gauthier and Bouchard's French-Canadian adaptation [51] of Spielberger's psychometric State-Trait Anxiety Inventory (STAI) questionnaire [52,53]. STAI was provided with permission from the copyright owner (Mind Garden, Inc [54]. Copyright prevents reproduction of the full scale). It is one of the most commonly used subjective measures of stress in health research, including emergency care [13,14,55-58]. This questionnaire is composed of two 20-item self-report subscales to measure 2 distinct anxiety concepts: (1) the temporary *state of anxiety* at the time of reporting (STAI form Y-1), which can be affected by stressful situations and 2) the more stable and long-standing presence of *trait anxiety* (form Y-2) [59]. Both forms can be used alone or as a complement. Form Y-1 was used in this study. To avoid interrupting the scenario and influence its veracity and inherent stress, no STAI was administered during the scenario. It was administered just before the scenario began to assess the stress at this moment, and again just after the end of the scenario by asking the paramedics to assess their maximum perceived stress during the scenario (Figure 2). Each item was mandatory to avoid missing values and answered on a 4-point Likert scale ranging from 1 (not at all) to 4 (very much). After reversing the scores for stress-absent items (ie, items 1, 2, 5, 8, 10, 11, 15, 16, 19, and 20) according to Spielberger’s instructions [59], the total score was calculated by summing up the weighted scores for the 20 items. STAI ranges from 20 to 80, with higher scores being positively correlated with greater stress [59]. A score greater than 40 is commonly used to define a clinical state of stress.

Perceived stress was also assessed by self-assessment using a numerical 10-point Likert visual analogue scale (VAS) [60]. Values ranged from 1 (totally unstressed) to 10 (totally stressed) to avoid neutral answers. To prevent any anticipation bias, participants were not informed that they would have to complete the STAI and VAS questionnaires after scenario completion (Figure 2). The questionnaires were provided onsite with the necessary precautions so that participants could not communicate with each other. No interaction other than detailing an item upon request of a participant occurred between the participants and investigators during the completion of the questionnaires.

#### Secondary Outcome: Physiologic Stress

HR was measured as a surrogate of the physiological sympathetic response to stress [61]. A single continuous measurement at 1-second interval was recorded during the scenario with optical photoplethysmography using a Polar A360 wrist-worn HR monitor (Polar Electro Oy). This wearable sensor has been previously validated for HR assessment [62,63], although it is not sensitive enough to track HR variability. The A360 was tightly attached to the participants’ wrist in accordance with the manufacturer’s specifications to avoid motion artifacts that could lead to inaccurate HR measurements. Data locally stored on the wristwatch itself during the scenarios was thereafter synchronized with the dedicated Polar FlowSync web service for later offline analysis. In line with previous research [64], several time-points of cardiovascular activity were measured: (1) the minimal HR measured within the 5 minutes before the scenario starts (HR_baseline_) while participants were not performing mental or physical exercise; (2) peak HR (HR_peak_) for each drug, defined as the maximal HR reached during the sequence from drug prescription by the physician to drug delivery; and (3) an additional HR_recovery_ was also measured as the minimal HR measured during the 5 minutes immediately following scenario completion (ie, at the stop of the timed period of the scenario represented by the patient’s arousal, but before debriefing).

### Data Collection

All scenarios were video-recorded by 3 GoPro Hero 5 and 7 Black Edition (GoPro Inc) video cameras mounted on participants and dispatched around them for later analysis. The camera setup was standardized. The investigators double-checked whether the questionnaires were fully and accurately completed. Data was collected using a REDCap database web app (REDCap, Vanderbilt University) hosted at Geneva University Hospitals and interfaced on an iPad Pro iOS 12.4 (Apple Inc). Neither follow up nor retention plans were required.

### Statistical Analysis

Assuming a two-sided α risk of .05, the number of participants enrolled in the parent study was sufficient to detect an effect size of 0.50, which corresponds to a medium effect of the app compared with the conventional methods on perceived stress (STAI-Y score), with a power of 80%. Outcomes with a single measurement per participant (including HR_peak_ per drug and postintervention STAI-Y and VAS) were compared between trial arms by using linear regression models with adjustment for the preintervention or baseline value and on centers to account for the randomized stratification. To compare the overall HR_peak_ between the arms, a linear regression model with mixed effects and adjusted for centers and HR_baseline_ was used to account for the multiple measures per participant (one per drug). With this model, the intercept was random with crossed random effects at the participant and drug levels. This model was adjusted for the centers and HR_baseline_. The Spearman correlation coefficient was used to assess the correlation between outcomes. Analyses were carried out using R software version 4.0.2, and the R package lme4 [65]. All statistical tests were two-sided, with an α risk of .05.

### Ethics Approval

This trial received a declaration of no objection by the Geneva Cantonal Ethics Committee and Swiss Ethics on March 29, 2018, as its purpose was to examine the effect of the intervention on health care providers. The study was conducted in accordance with the principles of the Declaration of Helsinki [66], Good Clinical Practice guidelines [67], the Consolidated Standards of Reporting Trials of Electronic and Mobile Health Applications and Online TeleHealth (CONSORT-EHEALTH) [68] guidelines, and the Reporting Guidelines for Health Care Simulation Research [69].

## Results

### Overview

**Figure 3:**
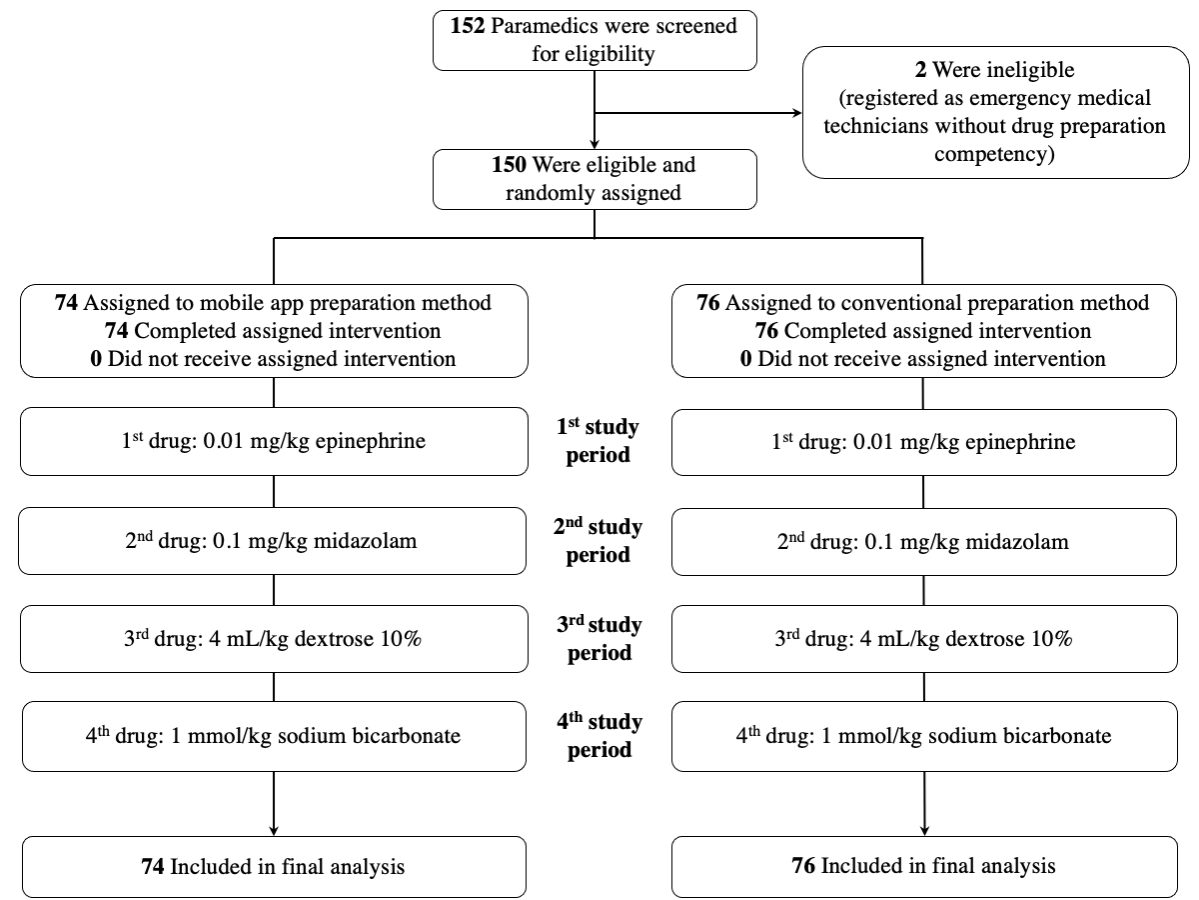
Screening, randomization, and analysis.

Of the 150 paramedics enrolled between September 3, 2019, and January 21, 2020, 74 were assigned to the intervention group and 76 to the control group. A total of 600 drug preparations were prepared. One participant's HR recording data were missing in the intervention group due to a technical problem with the watch. No dropouts occurred (Figure 3). The baseline characteristics are detailed in the published parent trial [38]. They were balanced in the 2 groups, and recruitment was balanced across the centers.

### Stress Response: Perceived Stress

**Figure 4:**
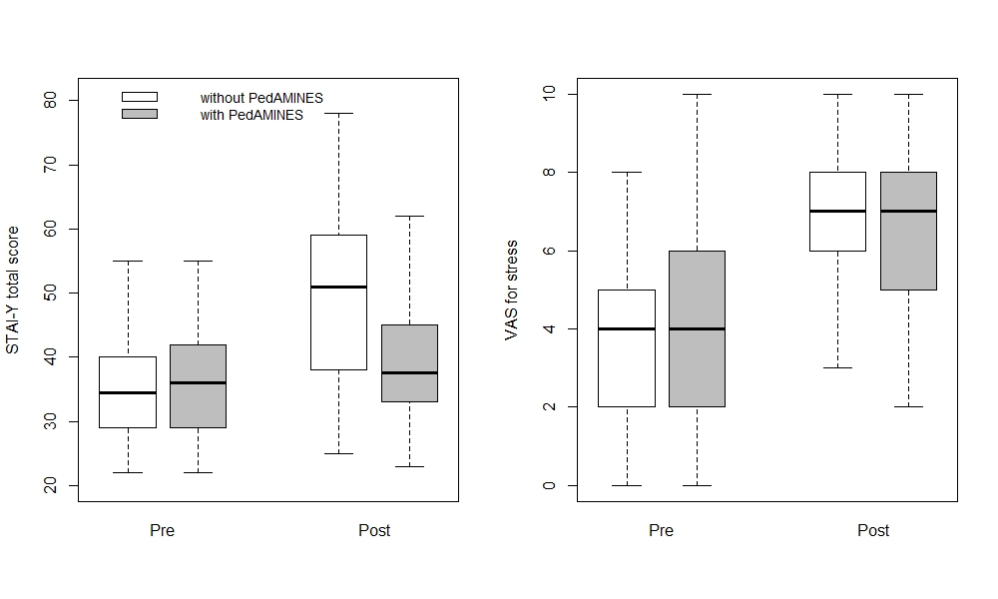
State-Trait Anxiety Inventory Form Y-1 and Visual Analogic Score box plots per study arm. PedAMINES: Pediatric Accurate Medication in Emergency Situations; STAI: State Trait Anxiety Inventory; VAS: visual analogue scale.

Both the STAI Form Y-1 and VAS questionnaires were completed by all 150 participants. Baseline STAI-perceived stress levels before the start of the scenario did not differ between the allocation groups (Table 1). The mean STAI-perceived stress scores of the participants supported by conventional methods then increased significantly at the time of drug preparations, whereas no significant increase was observed with the app support during the scenario. A higher stress increase was observed during the scenario using the conventional methods than the app (*P*<.001; Table 1 and Figure 4).

Similarly, on the VAS questionnaire, participants rated the mean perceived stress before the scenario as not different with or without the app support (Table 1 and Figure 4). After scenario completion, they reported a higher increase in stress at the peak of the scenario using the conventional method than the app. The paramedics’ gender, age, and years of experience did not modify the intervention effect, although for paramedics with more than 10 years of experience, the effect of the app seems to weaken (Multimedia Appendix 1).

**Table 1:** Comparison of STAI Form Y-1 and VAS scores before and after scenario completion (N=150).

		Mobile app (n=73), mean (SD)	Conventional method (n=76), mean (SD)	Difference (95% CI)^a^	*P* value
**STAI^b^**					
	Preintervention	36.1 (8.1)	35.4 (8.2)	0.8 (−1.7 to 3.3)	.55
	Postintervention	39.0 (8.4)	49.8 (13.2)	−11.4 (−14.7 to −8.0)	<.001
	Relative change (% of preintervention)	12.3 (29)	43.1 (35)	−30.1 (−39.8 to −20.5)	<.001
**VAS^c^**					
	Preintervention	4.2 (2.5)	3.9 (2.2)	0.3 (−0.4 to 1.0)	.35
	Postintervention	6.4 (1.9)	7.1 (1.8)	−0.8 (−1.3 to −0.2)	.006

^a^Linear regression models adjusted for center; in addition, differences in postintervention and relative change were adjusted for the preintervention values.

^b^STAI: State-Trait Anxiety Inventory.

^c^VAS: visual analogue scale.

### Stress Response: Heart Rate

**Figure 5:**
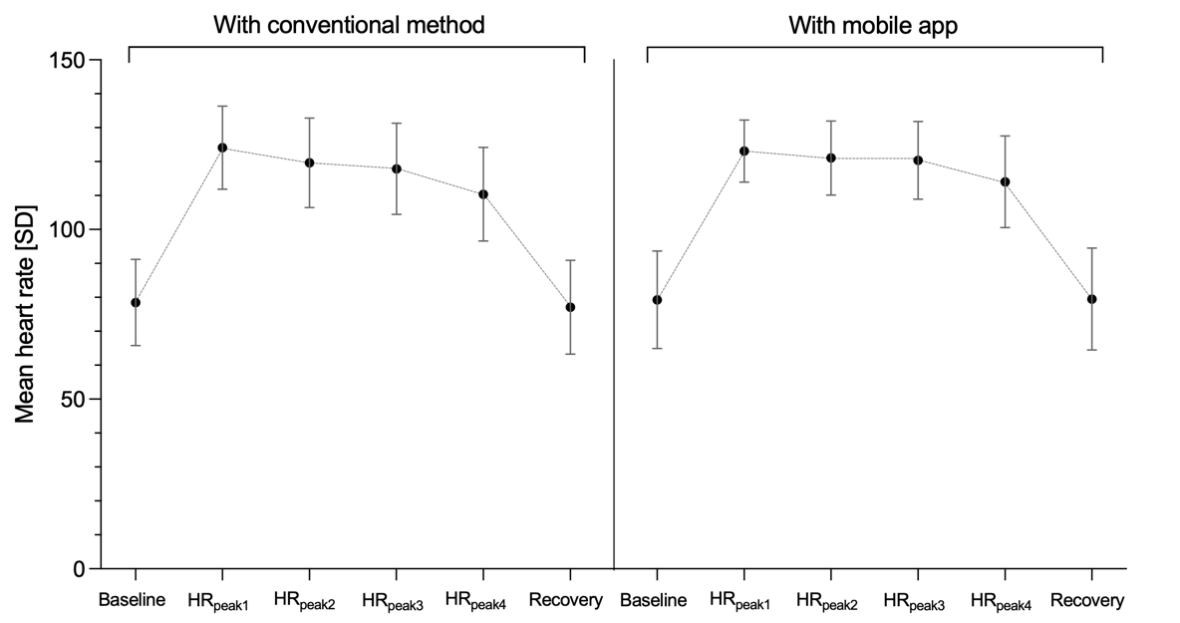
Mean heart rate (error bars=SD) in the baseline, the 4 consecutive heart rate peaks numbered according to the sequential prescription of each drug, and recovery time points over the course of the scenario. HR: heart rate.

The mean HR_baseline_ before the scenario started was approximately 80 beats per minute and similar between the allocation groups (Table 2). Maximal HR achieved during cardiac compressions was 122.5 (95% CI 118-127) in the app group and 123 (95% CI 117-128) in the control group and decreased in both groups before the drug preparation phase. During the scenario, the overall HR_peaks_ for the 4 drugs increased to 118 (95% CI 111 to 125) beats per minute with conventional methods and to 119.7 (95% CI 115 to 124) beats per minute with the app. This increase in HR from baseline represented approximately 50% of the mean HR_baseline_ (Figure 5). The difference between groups in HR_peaks_ during the scenario and over the 4 drugs was 1.4 (95% CI −1.8 to 4.6; *P*=.43) beats per minute, which was not statistically significant. The same was true for each drug (Table 2). After completion of the scenarios, HR declined to recovery values similar to HR_baseline_ in both groups.

**Table 2:** Heart rates before, during, and after scenario completion, per study group (N=150).

	Heart rate (bpm^a^)			
	Mobile app (n=73), mean (SD)	Conventional method (n=76), mean (SD)	Difference (95% CI)^b^	*P* value
Baseline	79.3 (14.4)	78.5 (12.7)	1.0 (−3.3 to 5.3)	.64
First drug, HR^c^_peak_	123.1 (9.2)	124.1 (12.2)	−1.1 (−4.7 to 2.4)	.53
Second drug, HR_peak_	121.1 (10.9)	119.9 (13.3)	1.0 (−2.8 to 4.8)	.61
Third drug, HR_peak_	120.4 (11.4)	117.9 (13.3)	2.4 (−1.4 to 6.2)	.22
Fourth drug, HR_peak_	114.1 (13.5)	110.5 (13.7)	3.4 (−0.9 to 7.6)	.12
Recovery	79.3 (15.0)	76.8 (13.7)	1.9 (−2.3 to 6.1)	.37
Maximal HR_peak_^d^	126.1 (10.3)	126.0 (12.1)	−0.1 (−3.8 to 3.5)	.95

^a^bpm: beats per minute.

^b^Linear regression models adjusted for center and baseline heart rate using linear regression models (except for baseline heart rate adjusted for center only). A negative difference means that the average value is lower with than without PedAMINES.

^c^HR: heart rate.

^d^Highest value of HR_peak_ among the 4 drugs.

### Correlation Between Perceived Stress and Heart Rate

A strong correlation was found between STAI-Y and the VAS scores for the perceived stress by the participants before the intervention (Spearman *r*=0.72) and after the intervention (Spearman *r*=0.68). However, the STAI-Y and the VAS scores before the intervention were poorly correlated with HR at baseline (Spearman *r*=0.14 and *r*=0.22, respectively). The STAI-Y and VAS scores after the intervention were not correlated with the maximal HR_peak_ (Spearman *r*=0.02 and *r*=−0.04, respectively)

## Discussion

### Principal Findings

The unpredictable out-of-hospital environment and high-stake CPR situations lead to major stressful experiences for involved rescuers, which can adversely affect patient safety [5]. In this randomized controlled trial, we report a significant increase in both perceived and physiological stress in paramedics during the preparation of emergency drugs for pediatric OHCA patients in a simulated model. However, perceived stress was 27% lower among those who used a specific mHealth app designed to facilitate drug preparation compared with those who only had access to conventional preparation methods. This result was observed irrespective of paramedics’ years of experience, age, or gender, suggesting a worthwhile benefit of its use in the prehospital setting.

Quantification of the acute stress response of rescuers during CPR has been the subject of many studies, although no single stress-specific marker for its measurement has been definitively validated [70]. Thus, previous studies have used, alone or in conjunction, different surrogate stress markers (ie, biological, electrophysiological or psychological) to assess the relationship between stress and CPR performance [11]. Those considering subjective stress markers by means of self-reported questionnaires, such as the STAI, showed the strongest association with lower performance [6,11]. On the other hand, stress-coping strategies such as leadership and stress management training, cognitive aids (eg, checklists and algorithms), and mindfulness meditation have been shown to enhance CPR performance [5,71]. However, these studies mostly focused primarily on residents [11], a less experienced and likely more stress-prone population, thus providing little evidence regarding more experienced rescuers. Evidence regarding paramedics is scarce. Few studies in the field have yielded conflicting results. LeBlanc et al [56] compared paramedics’ acute perceived and salivary cortisol stress responses and performance during simulated low- and high-stress scenarios. They observed impairments in some aspects of clinical performance in response to a high-stress scenario. Among these aspects, stress significantly impaired drug dosage calculation and was associated with a greater risk of error [14]. Interestingly, these authors reported a subjective mean STAI-rated overall stress of 46.1 for paramedics under high-stress conditions, corroborating our own findings. Conversely, Bjørshol et al [72] observed no performance impairment associated with higher self-reported stress in a randomized trial comparing paramedics under calm CPR conditions with those under stressful CPR conditions. Unfortunately, in these studies, no evaluation of interventions aimed at specifically reducing stress during CPR and studying their consecutive impact on performance was carried out. To date, the bulk of the research and development for interventions to reduce stress has mostly focused on long-lasting mental illnesses using mobile self-management apps in the field of health psychology [73]. To our knowledge, this trial is the first of its kind to report reduced perceived stress during multiple drug dosage calculations by advanced care paramedics supported by a mobile app in acute life-threatening situations. Prehospital dosing errors, although probably underreported due to failure to recognize them or reluctance to report them [70,74], affect approximately 56,000 children treated by EMS each year in the United States, with drugs administered outside of the proper dose range reported in up to 39.8% of more than 5500 children [31,75]. Facilitating emergency drug preparation at pediatric dosage while reducing stress might reduce these errors.

Acute stress situations elicit not only a psychological but also an adaptive, generally transient, physiological stress response carried out by regulatory pathways through the activation of the cardiovascular, endocrine, immune, and autonomic nervous systems [76,77]. The overall stress response is a combination of these complex and relatively independent pathways, without necessarily showing correlations with each other [57,58,78]. Additionally, there are interindividual differences in perceived and physiological stress responses in the same situation [76,78]. In this study, although perceived acute stress was reduced by the use of the app, such a reduction was not observed in HR that remained high in both groups during the scenario. This finding is consistent with that of the existing literature. At the individual level, the relationship between self-reported perceived stress and physiological stress measured by objective parameters such as HR has been shown to be somewhat inconsistent [57,79]. Clarke et al [80] examined the relationship between emergency medicine residents’ self-reported stress before and after a simulation exercise and HR throughout the scenario. They observed that HR elevation alone correlated poorly with both perceived stress and clinical performance. Similar results were observed in another study where varying stress levels in simulated trauma scenarios elicited higher subjective stress and cortisol levels and poorer performance among residents exposed to high-stress conditions, whereas HR elevation was not significantly different between low- or high-stress conditions [81]. Among the reasons that may explain this phenomenon, it has been speculated that strenuous physical activity could be a confounding factor for HR, limiting its value as a marker of mental stress in acute situations [6,11]. In this study, physical activity was limited to the initial hands-on resuscitation period. Thereafter, physical activity was restricted to the preparation of drugs in close proximity to the patient, thus limiting exertion. Hence, although indicative of a state of mental stress in the absence of physical exertion, it appears from this study that sustained HR at high levels cannot be used alone as a physiological expression of perceived stress. An alternative explanation for the variation in HR observed over the course of the scenario may lie in the level of stress-induced physiological activation desirable for optimal task performance, with higher HR levels observed during drug preparations. This is consistent with the relationship between stress and performance that has been described theoretically by Yerkes-Dodson [82] and follows an inverted U-shaped curve. At low levels of stress, performance is poor but increases with increasing stress levels until a certain optimal point is reached, beyond which task performance and decision making abilities could become impaired, followed by a decline in performance quality [83]. Although we cannot comment on whether the observed HR levels represented the optimal HR zone for best performance, our results suggest that an adaptive physiological response to stress, at least in terms of HR levels, was similarly triggered in both study groups.

### Limitations

This study had several limitations. First, as drug preparation times varied considerably from one participant to another and could have influenced any stress attenuation or exaggeration over time, we decided not to measure average HRs per drug. Second, the collection of additional physiological stress markers was not carried out, which could also be seen as a limitation. However, measuring paramedics’ cortisol levels would have required complex consideration of intra and interindividual circadian rhythms among day and night shift paramedics, as well as serial collection of blood, saliva, or sweat samples to capture baseline and in-scenario values, making the procedure impractical and potentially prone to preparation bias [84]. On the other hand, measuring paramedics’ HR variability, which has been shown to be influenced by stress during emergency care and a reliable means of monitoring it [11,57,85], would require signal acquisition by an electrocardiogram. This could have potentially restrained paramedics' movements and involvement, thus hindering their ability to prepare the drugs according to usual practice. Considering the use of wearable photoplethysmography to measure pulse rate variability may be a promising alternative to overcome this limitation in future studies [86,87]. Another limitation of our trial includes its simulated setting, which raises the concern of its generalizability to real-life situations. High-fidelity simulation has been shown to be as realistic and stressful as real-life situations, both at the psychological and physiological levels [6,11,88]. Furthermore, simulation is an essential method for assessing research questions and technologies that cannot be answered during real CPR as the diversity among patients and their diseases makes such studies difficult to standardize in critical situations [89]. In addition, in the voluntary absence of correlational analysis in this study due to a nonexhaustive investigation of all stress response axes, our data does not allow causal inferences to be made about the relationship between psychological and physiological stress reactivity and emergency drugs preparation. They only provide a sketch in this direction, which requires further research. Finally, we acknowledge that this study did not distinguish whether and to what extent emergency drugs preparation had a greater influence on stress than the pediatric OHCA situation itself or vice versa.

### Conclusions

In this nested randomized controlled trial, paramedics confronted with a high-stress pediatric OHCA scenario reported a lower level of perceived stress when using a mobile app designed to help pediatric drug preparation compared with conventional calculation methods. As acute perceived stress is associated with lower CPR performance in calculating drug dosages and as the children are particularly vulnerable to such errors, we suggest that dedicated medical mobile apps have the potential to reduce stress while improving medication safety. This could change the prehospital clinical practice in the area of pediatric emergency medicine. The next step would be to determine in real-life studies whether stress reduction through the app translates into improved clinical outcomes to contrast the results of this actual research.

## Appendix

Multimedia Appendix 1 Subgroup analysis for the primary outcome by gender, age, and years since paramedic certification and State-Trait Anxiety Inventory perceived stress score.

Multimedia Appendix 2 PedAMINES Prehospital Trial Group.

## Abbreviations

CPR: cardiopulmonary resuscitation
EMS: emergency medical services
mHealth: mobile health
OHCA: out-of-hospital cardiac arrest
PedAMINES: Pediatric Accurate Medication in Emergency Situations
STAI: State-Trait Anxiety Inventory
VAS: visual analogue scale
