# Supplementary material for: Impact of a Mobile App on Paramedics’ Perceived and Physiologic Stress Response During Simulated Prehospital Pediatric Cardiopulmonary Resuscitation: Study Nested Within a Multicenter Randomized Controlled Trial
Source: JMIR Mhealth Uhealth. 2021 Oct 7;9(10):e31748. doi: 10.2196/31748 (PMC8532016; doi:10.2196/31748)
Supplement: Multimedia Appendix 1 [file mhealth_v9i10e31748_app1.docx]

|  | Mobile app (n=73)  mean (SD) | Conventional method (n=76)  mean (SD) | Difference (95% CI)^a^ | *P* value |
| --- | --- | --- | --- | --- |
| STAI, female |  |  |  | |
| post-intervention | 39.0 (+/-7.8) | 55.9 (+/-11.4) | -16.0 (-22.3 to -9.7) | <.001 |
| relative change (% of pre) | 6.2 (+/-28.2) | 48.3 (+/-36.2) | -41.6 (-58.9 to -24.4) | <.001 |
| STAI, male |  |  |  |  |
| post-intervention | 38.9 (+/-8.8) | 47.2 (+/-13.1) | -9.2 (-13.5 to -5.0) | <.001 |
| relative change (% of pre) | 15.0 (+/-29.2) | 40.8 (+/-34.6) | -24.8 (-37.3 to -12.2) | <.001 |
| STAI, age < 34 years |  |  |  |  |
| post-intervention | 39.3 (+/-8.4) | 52.0 (+/-12.9) | -13.7 (-19.1 to -8.3) | <.001 |
| relative change (% of pre) | 8.9 (+/-27.3) | 48.3 (+/-30.2) | -35.6 (-50.9 to -20.2) | <.001 |
| STAI, age > 34 years |  |  |  |  |
| post-intervention | 38.7 (+/-8.5) | 48.0 (+/-13.3) | -10.0 (-14.8 to -5.2) | <.001 |
| relative change (% of pre) | 13.8 (+/-30.1) | 38.7 (+/-38.5) | -27.5 (-41.6 to -13.3) | <.001 |
| STAI, experience < 5 years |  |  |  |  |
| post-intervention | 38.3 (+/-9.6) | 49.4 (+/-12.0) | -12.8 (-19.0 to -6.6) | <.001 |
| relative change (% of pre) | 9.6 (+/-27.0) | 44.9 (+/-34.1) | -35.3 (-53.6 to -17.0) | <.001 |
| STAI, experience 5-10 years |  |  |  |  |
| post-intervention | 38.8 (+/-7.8) | 55.0 (+/-12.4) | -16.3 (-22.5 to -10.0) | <.001 |
| relative change (% of pre) | 12.8 (+/-29.8) | 55.0 (+/-38.3) | -46.0 (-64.4 to -27.6) | <.001 |
| STAI, experience >10 years |  |  |  | |
| post-intervention | 40.2 (+/-7.9) | 44.6 (+/-13.7) | -5.8 (-13.3 to 1.8) | .13 |
| relative change (% of pre) | 13.4 (+/-31.6) | 27.6 (+/-26.9) | -13.3 (-32.8 to 6.21) | .17 |
