# Supplementary material for: Impact of a Mobile App on Paramedics’ Perceived and Physiologic Stress Response During Simulated Prehospital Pediatric Cardiopulmonary Resuscitation: Study Nested Within a Multicenter Randomized Controlled Trial
Source: JMIR Mhealth Uhealth. 2021 Oct 7;9(10):e31748. doi: 10.2196/31748 (PMC8532016; doi:10.2196/31748)
Supplement: Multimedia Appendix 2 [file mhealth_v9i10e31748_app2.docx]

**PedAMINES Prehospital Trial Group**

**Principal investigators**

- **Geneva University Children’s Hospital, Geneva, Switzerland**

Johan N Siebert, Laurie Bloudeau, Christophe Combescure, Kevin Haddad, Florence Hugon, Laurent Suppan, Frédérique Rodieux, Christian Lovis, Alain Gervaix, Frédéric Ehrler, Sergio Manzano

**Collaborators and on-site study coordinators**

- **A.C.E. Genève Ambulances SA, Geneva, Switzerland**

Laurie Bloudeau, EMS-p

- **Geneva Team Ambulances (GTA), Geneva, Switzerland**

Marec Saillant, EMS-p

- **SK Ambulances, Geneva, Switzerland**

Renaud Grandjean, EMS-p

- **Secours Ambulances Genève (SAG), Geneva, Switzerland**

Annick Leuenberger, EMS-p

- **Service de Sauvetage et de Lutte contre les Incendies Aéroportuaires (SSLIA), Geneva, Switzerland**

Pascal Donnet, , EMS-p; Philippe Hauck, EMS-p

- **Service d'Incendie et de Secours (SIS), Geneva, Switzerland**

Sébastien Pappalardo, EMS-p

- **Ambulance Riviera, La Tour-de-Peilz, Switzerland**

Philippe Nidegger, EMS-p

- **Air Zermatt, Zermatt, Switzerland**

David Neel, Flight EMS-p

- **Höhere Fachschule für Rettungsberufe (HFRB), Zürich, Switzerland**

Stephan Steinhauser, MD

- **Servizio Ambulanza Locarnese e Valli (SALVA), Ticino, Switzerland**

Michel Ceschi, EMS-p; Bruno Belli, EMS-p

- **Ambulances du Sud Fribourgeois, Vaulruz, Switzerland**

Sébastien Ottet, EMS-p; Wenceslao Garcia, MD

- **Service Communal de la Sécurité (SCS), Neuchâtel, Switzerland**

Yoan Mollier, EMS-p; Yves Vollenweider, EMS-p ; Pierre Voumard, EMS-p

- **Service de Protection et Sauvetage Lausanne (SPSL), Lausanne, Switzerland**

Karine Corbat, EMS-p; Philippe Robadey, EMS-p

- **Centre de Secours et d'Urgences (CSU) Morges-Aubonne, Aubonne, Switzerland**

Joël Bauer, EMS-p; Cyril Berger, EMS-p
